# Antismoking Media Campaign and Smoking Cessation Outcomes, New York State, 2003-2009

**Published:** 2012-01-19

**Authors:** Kevin C. Davis, Matthew C. Farrelly, Jennifer Duke, Lisa Kelly, Jeffrey Willett

**Affiliations:** RTI International; RTI International, Research Triangle Park, North Carolina; RTI International, Research Triangle Park, North Carolina; New York State Department of Health, Tobacco Control Program, Albany, New York; New York State Department of Health, Tobacco Control Program, Albany, New York

## Abstract

**Introduction:**

The New York Tobacco Control Program (NY TCP) is one of the largest state tobacco control programs in the United States. Little research has been published on the effectiveness of its antismoking media campaign. The objective of this study was to examine whether exposure to NY TCP's statewide antismoking media campaign corresponded to smoking outcomes.

**Methods:**

We used data from the 2003 through 2009 New York Adult Tobacco Survey to evaluate exposure to NY TCP advertising, cessation intentions, quit attempts, and cigarette consumption among New York adult smokers. We also used data from the 2003 through 2009 New York Behavioral Risk Factor Surveillance System (BRFSS) and the 2003 through 2009 National Health Interview Survey (NHIS) to examine smoking prevalence among New York adults compared with US adults.

**Results:**

From 2003 through 2009, smokers' exposure to NY TCP advertising increased from 6% to 45%, the prevalence of 30-day intentions to quit increased from 26% to 35%, and the prevalence of quit attempts increased from 46% to 62%. Average cigarettes smoked per day decreased from 15 in 2003 to 11 in 2009. The New York BRFSS and NHIS both showed significant downward trends in adult smoking prevalence. The decline during this period was greater in New York (18%) than in the United States as a whole (5%).

**Conclusion:**

NY TCP's campaign generated significant increases in exposure to advertising over time that corresponded with changes in key cessation- and smoking-related outcomes. Findings suggest that NY TCP's sustained implementation of evidence-based cessation advertisements contributed to these changes.

## Introduction

Statewide tobacco control programs have contributed to significantly decreased smoking prevalence and cigarette consumption among adults and youth in the United States (1-7). The success of early tobacco control programs in California (8), Massachusetts (9), and Florida (10) served as examples to other states implementing their own tobacco control programs that included multimedia antismoking campaigns. Mass media campaigns can be effective in promoting smoking-related behavior change (11).

The New York Tobacco Control Program (NY TCP) is one of the largest statewide tobacco control programs in the United States. Since 2003, NY TCP has invested approximately $75 million in paid advertising on television, radio, print, the Internet, and other venues to educate New York residents about the health risks of smoking and the dangers of secondhand smoke exposure. The program's health communication interventions consist primarily of paid media campaigns aimed at encouraging smokers to quit. On the basis of evidence supporting "intense" advertisements, defined as using graphic images to depict the health consequences of smoking or emotional portrayals of personal loss and smoking-related hardships (5,12), NY TCP focused resources on airing advertisements with strong graphic and emotional content. Starting in 2005, NY TCP devoted 70% of its resources for paid media to intense advertisements, such as the "Pam Laffin" series, (which features a young mother who developed fatal emphysema as a result of smoking at a young age and portrays the resulting emotional hardship of her children) and the "Every Cigarette Does Damage" series from Australia (which uses graphic images to illustrate the health effects of smoking). NY TCP complemented these messages with advertisements intended to increase smokers' self-efficacy to quit. These advertisements typically provided smokers with information and resources on how to quit, including specific websites or quitlines.

These series were examples of NY TCP's strategy to use existing advertisements, drawn primarily from the Centers for Disease Control and Prevention (CDC) Media Campaign Resource Center and from other countries. The cost savings of this strategy enabled the program to invest more resources in the placement and purchase of advertising time rather than the development of new creative material. Meanwhile, NY TCP's budget for paid advertising also grew — from $8 million in fiscal year (FY) 2004 (April 1, 2004, to March 31, 2005) to $12 million in FY 2006 to $19 million in FY 2009.

Advertisements from NY TCP's media campaign were responsible for significant increases in calls to the New York Smokers' Quitline (13). However, no published studies have examined whether the campaign corresponded with other key outcomes such as intentions to quit and quit attempts. The objective of this study was to examine the extent to which New Yorkers were exposed to the campaign and whether trends in campaign exposure corresponded with trends in key outcomes (ie, cessation attempts and intentions, cigarette consumption, and overall prevalence) over time.

## Methods

### New York Adult Tobacco Survey

We used data from the New York Adult Tobacco Survey (ATS), which was fielded quarterly from June 2003 through December 2009 (14). The ATS is a random-digit–dialed telephone survey designed to reach representative samples of New York State adults aged 18 years or older. The ATS questionnaire measures attitudes, beliefs, intentions, behaviors, and other NY TCP outcomes regarding cessation and secondhand smoke. The ATS also measures exposure to NY TCP activities and services, including antismoking advertising. Most ATS measures we analyzed are key outcome indicators from CDC (15). Our analyses are limited to New York adult smokers (N = 8,608). The survey data are weighted to reflect the state population of adults, adjusting for different probabilities of selection and survey nonresponse. The institutional review boards of RTI International and the New York State Department of Health approved all ATS survey protocols.

### National comparison data

We examined data from the New York Behavioral Risk Factor Surveillance System (BRFSS) and the National Health Interview Survey (NHIS) from 2003 through 2009 to compare trends in current smoking prevalence between New York and the United States as a whole. BRFSS is sponsored by CDC and is administered by individual states to collect state-specific data on health outcomes and risk factors, including tobacco use (16). NY TCP uses BRFSS for its official published estimates of adult smoking prevalence in New York. NHIS, also sponsored by CDC, is a cross-sectional household interview survey that provides nationally representative data on health conditions and health risk factors (including tobacco use) (17). Both BRFSS and NHIS data are weighted to reflect census population totals for age, race/ethnicity, and sex and are adjusted for probabilities of selection and survey nonresponse.

### Measures

**Exposure to paid advertising**

We assessed exposure to NY TCP paid advertising with both individual-level, self-reported measures of awareness in the ATS and market-level measures of advertising gross ratings points (GRPs). The ATS asks about specific campaign television advertisements that aired either at the time of or approximately 6 weeks before the interview. Respondents were first asked whether they had "recently seen an antismoking advertisement on TV that shows . . ." followed by a brief description of the beginning of the advertisement. Respondents who indicated initial recognition in this first question were then asked to provide further details about what happens in the advertisement. Respondents who accurately described the advertisement were considered to have confirmed recall. We then used a dichotomous indicator variable to define overall campaign recall as having confirmed awareness of at least 1 advertisement from the campaign. This is a validated method of measuring confirmed advertisement recall and has been used in numerous other evaluation studies of antismoking campaigns (11,18-21).

We measured market-level exposure to NY TCP paid advertising with quarterly population-weighted GRP data compiled by the campaign's media buyer. GRPs are based on television ratings for programs on which the campaign advertisements aired to give the relative "dose" of the campaign in each media market in New York. The ratings data provide estimates of the percentage of the audience that watched the programs (also known as "reach") and the frequency with which they aired. Because the television ratings are averages of audience reach and exposure frequency in a given market, GRPs represent the respondent's potential exposure to the campaign. Market-level GRPs have also been used in several other studies to evaluate the effect of antismoking media campaigns (20,22-24).

**Cessation and smoking-related outcomes**

The ATS measures intentions to quit smoking with 2 items that assess whether the smoker intends to make a quit attempt in the next 30 days and whether the smoker is seriously considering quitting smoking in the next 6 months. Past-year quit attempts are measured using an indicator for whether the smoker had stopped smoking for 1 day or longer in the past 12 months in an attempt to quit smoking. Cigarette consumption was measured as the average number of cigarettes smoked per day among current smokers. Lastly, current smoking status (defined as smoking at least 100 cigarettes in lifetime and now smoking some days or every day) was measured in both the New York BRFSS and NHIS in the 2003 through 2009 surveys.

### Statistical analysis

We first assessed long-term trends in New Yorkers' exposure to campaign advertisements by examining self-reported recall of advertisements among smokers and market-level GRPs by advertisement type. We then summarized corresponding trends in key tobacco use-related outcomes over time. For each outcome indicator, we performed trend tests (bivariate regressions between the outcome indicator and time) to determine whether each trend was significant at *P* < .05. We used Stata version 11 (StataCorp LP, College Station, Texas) to conduct all analyses.

## Results

### Exposure to paid advertising

**Figure 1.**
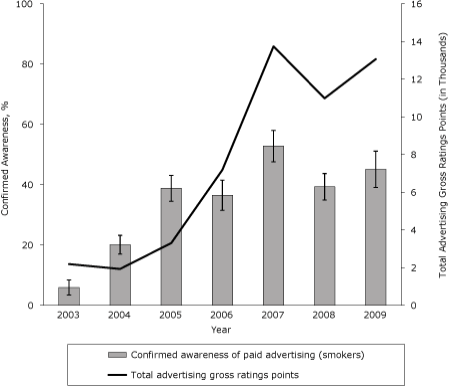
Confirmed awareness of antismoking media campaign advertisements among smokers in New York State, 2003-2009. Advertising gross ratings points are a market-level aggregate measure of campaign reach and frequency, expressed in thousands. Confirmed awareness represents the proportion of adult smokers who indicated awareness of any New York Tobacco Control Program advertisement and is based on self-reported data in the New York Adult Tobacco Survey, 2003-2009 (N = 8,608). Vertical lines within bars represent 95% confidence intervals (CIs)

Confirmed awareness of NY TCP advertisements among New York smokers increased from 6% in 2003 to 45% in 2009 (Figure 1) (β, 0.07; SE, 0.007; *P* < .001). However, awareness peaked at 53% in 2007 and declined to 39% in 2008, corresponding with concurrent drops in paid advertising. Confirmed awareness rebounded again in 2009 to 45% as the campaign was back on air consistently during the first half of the year.

**Table:** 

**Year**	**Total Advertising Gross Ratings Points (in Thousands)**	**Confirmed Awareness, % (95% CI)**
2003	2.186	5.9 (3.4-8.4)
2004	1.928	20.1 (16.9-23.2)
2005	3.304	38.8 (34.5-43.0)
2006	7.185	36.5 (31.4-41.5)
2007	13.742	52.8 (47.6-58.0)
2008	10.985	39.3 (34.9-43.7)
2009	13.065	45.1 (39.6-50.6)

### Trends in smoking-related outcomes

**Figure 2.**
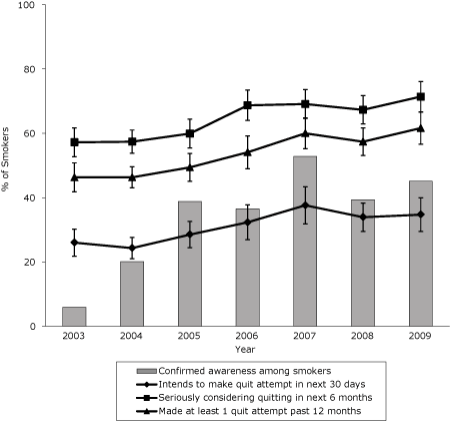
30-day and 6-month intentions to quit and past-year quit attempts among smokers in New York State, 2003-2009. Confirmed awareness represents the proportion of adult smokers who indicated awareness of any New York Tobacco Control Program advertisement. Confirmed awareness, 6-month quit intentions, 30-day quit intentions, and past 12-month quit attempts are based on self-reported data in the New York Adult Tobacco Survey, 2003-2009 (N = 8,608). Vertical lines within bars represent 95% confidence intervals (CIs).

The percentage of smokers who intend to make a quit attempt in the next 30 days increased from 26% in 2003 to 35% in 2009 (Figure 2) (β, 0.03; SE, 0.006; P < .001). Similarly, the percentage of smokers who were seriously considering quitting smoking in the next 6 months increased from 57% in 2003 to 71% in 2009 (β, 0.03; SE, 0.008; P < .001).

**Table:** 

**Year**	**Confirmed Awareness, % (95% (CI)**	**Seriously Considering Quitting in Next 6 Months, % (95% CI)**	**Intends to Make Quit Attempt in Next 30 Days, % (95% CI)**	**Made at Least 1 Quit Attempt Past 12 Months, % (95% CI)**
2003	5.9 (3.4-8.4)	57.2 (52.6-61.7)	26.0 (21.8-30.2)	46.3 (41.9-50.8)
2004	20.1 (16.9-23.2)	57.4 (53.8-61.0)	24.3 (21.0-27.5)	46.3 (43.0-49.6)
2005	38.8 (34.5-43.0)	59.9 (55.4-64.4)	28.5 (24.4-32.6)	49.4 (45.1-49.6)
2006	36.5 (31.4-41.5)	68.7 (63.9-73.4)	32.3 (26.9-37.7)	54.1 (45.1-53.8)
2007	52.8 (47.6-58.0)	69.1 (64.6-73.6)	37.6 (31.8-43.4)	60.0 (55.2-64.8)
2008	39.3 (34.9-43.7)	67.3 (62.9-71.6)	33.9 (29.5-38.3)	57.4 (53.1-61.8)
2009	45.1 (39.6-50.6)	71.4 (66.7-76.2)	34.7 (29.5-39.9)	61.6 (56.7-66.6)

**Figure 3.**
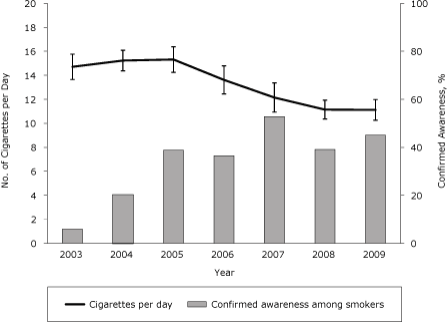
Cigarette consumption among smokers in New York, 2003-2009. Confirmed awareness represents the proportion of adult smokers who indicated awareness of any New York Tobacco Control Program advertisement. Both confirmed awareness and cigarettes per day are based on self-reported data in the New York Adult Tobacco Survey, 2003-2009 (N = 8,608). Vertical lines within bars represent 95% confidence intervals (CIs).

The percentage of smokers who made at least 1 quit attempt in the past 12 months increased from 46% in 2003 to 62% in 2009 (β, 0.03; SE, 0.005; *P* < .001) (Figure 2). Cigarette consumption among smokers in New York also declined significantly during this period (Figure 3). The average number of cigarettes smoked per day decreased from 15 in 2003 to 11 in 2009, a decline of nearly 25% (β, −0.22; SE, 0.029; *P* < .001).

**Table:** 

**Year**	**Confirmed Awareness, % (95% CI)**	**No. of Cigarettes Per Day (95% CI)**
2003	5.9 (3.4-8.4)	14.7 (13.7-15.8)
2004	20.1 (16.9-23.2)	15.2 (14.4-16.1)
2005	38.8 (34.5-43.0)	15.3 (14.3-16.4)
2006	36.5 (31.4-41.5)	13.6 (12.4-14.8)
2007	52.8 (47.6-58.0)	12.1 (10.9-13.4)
2008	39.3 (34.9-43.7)	11.1 (10.4-11.9)
2009	45.1 (39.6-50.6)	11.1 (10.3-11.9)

**Figure 4.**
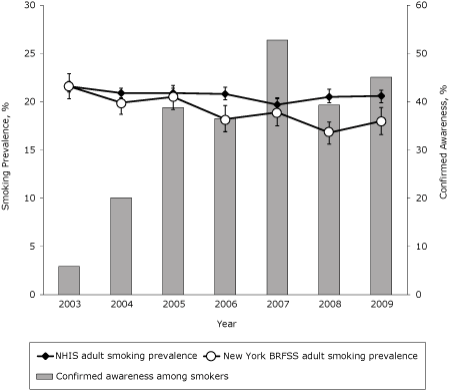
Smoking prevalence among New York adults compared with US adults, 2003-2009. Confirmed awareness represents the proportion of adult smokers who indicated awareness of any New York Tobacco Control Program advertisement and is based on self-reported data in the New York Adult Tobacco Survey, 2003-2009. Adult smoking prevalence is defined as smoking at least 100 cigarettes in lifetime and now smoking some days or every day. Smoking prevalence estimates are generated separately from the National Health Interview Survey (NHIS) (N = 188,637) and the New York Behavioral Risk Factor Surveillance System (BRFSS) (N = 46,315), 2003-2009. Vertical lines within bars represent 95% confidence intervals (CIs).

Smoking prevalence decreased nationally from 22% in 2003 to 21% in 2009 (Figure 4), a decline of 5% (β, −0.009; SE, 0.003; P = .005). Smoking prevalence among New York adults declined more steeply from 22% in 2003 to 18% in 2009, a decline of 18% (β, −0.043; SE, 0.008; P < .001).

**Table:** 

**Year**	**Confirmed Awareness, % (95% CI)**	**NHIS Adult Smoking Prevalence, % (95% CI)**	**New York BRFSS Adult Smoking Prevalence, % (95% CI)**
2003	5.9 (3.4-8.4)	21.6 (21.0-22.1)	21.6 (20.3-22.9)
2004	20.1 (16.9-23.2)	20.9 (20.4-21.4)	19.9 (18.7-21.1)
2005	38.8 (34.5-43.0)	20.9 (20.4-21.4)	20.5 (19.2-21.7)
2006	36.5 (31.4-41.5)	20.8 (20.2-21.5)	18.2 (16.9-19.6)
2007	52.8 (47.6-58.0)	19.7 (19.1-20.4)	18.9 (17.5-20.3)
2008	39.3 (34.9-43.7)	20.5 (19.9-21.3)	16.8 (15.6-17.9)
2009	45.1 (39.6-50.6)	20.6 (19.9-21.2)	18.0 (16.6-19.4)

## Discussion

NY TCP's antismoking media campaign resulted in significant increases in smokers' exposure to paid advertisements. Concurrent with these increases, both intentions to quit and cessation attempts increased significantly among smokers from 2003 through 2009, and current smoking prevalence significantly declined, consistent with similar findings in many other state campaign evaluations (11). On the basis of New York State adult population estimates in 2003 and 2009, these results suggest that there were approximately 502,000 fewer current smokers and 204,000 more smokers trying to quit in 2009 than in 2003.

Although many outcomes changed favorably during this time, many of these changes appeared to plateau during 2008 and 2009. This plateau in outcome change corresponded with declines in advertising activity in 2008 and the latter half of 2009. Positive changes in cessation-related outcomes between 2007 and 2009 were minimal compared to prior yearly changes from 2003 to 2006. These patterns suggest that fluctuations in advertising efforts affect both individual-level exposure to the campaign and trends in key outcome indicators, underscoring that consistent implementation is necessary for the long-term success of health communication campaigns.

Several lessons can be drawn from NY TCP's implementation of health communication interventions that may be instructive for other states. First, increases in media expenditures and placements directly translate into increases in individual awareness of advertisements. In addition, heavy resources are required to attain measurable levels of exposure to campaign messages. Since 2003, NY TCP's budget for health communication increased dramatically, which translated into significant increases in smokers' awareness of the campaign. Maintaining high levels of exposure is necessary to effect change (25) but is a challenge because of uncertainties about the availability of funds for paid advertising.

A second lesson is that NY TCP's media campaign used off-the-shelf advertisements that are evidence-based and readily available from CDC's Media Campaign Resource Center and other outlets. This choice allowed the campaign to avoid costly formative and creative content development and devote more resources to media placement and advertising purchases. The net result was that NY TCP was able to maximize smokers' awareness of the campaign and influence key outcomes with existing media resources.

This study had several limitations. First, NY TCP's media campaign occurred in the context of other broader program efforts including community partnerships, policy promotion, and cessation services such as the New York State Smokers' Quitline and cessation centers. In addition, antismoking policies changed during this period and included a dramatic increase in cigarette excise taxes in 2009, which may have helped to offset the effect of reductions in campaign GRPs that occurred between 2007 and 2009. These factors can make it difficult to identify the effects of any single program area, including media. A second limitation is that our data are limited to New York adult smokers and thus may not be generalized to the national population of smokers.

However, 2 aspects of the media campaign highlight its plausibility as a driver in the observed outcome changes. First, the NY TCP media campaign has consistently been one of the largest single interventions in the program, representing more than 25% of the overall NY TCP budget over the life of the program. Second, the media campaign also likely represents the program's most influential intervention in terms of the overall number of smokers reached, because of the wide distribution and visibility of television advertisements. To confirm the influence of the campaign, future studies using multivariate models to examine the direct relationship between exogenous market-level campaign GRPs and individual-level outcome indicators are needed. These future studies should undertake analyses to identify the specific effects of the media campaign, adjusting for other potentially confounding influences.
